# CSF NPTXs sensitively monitor neurodegeneration and cognitive decline in the Alzheimer's disease continuum

**DOI:** 10.1002/alz70856_102439

**Published:** 2025-12-24

**Authors:** Linbin Dai, Feng Gao, Yong Shen

**Affiliations:** ^1^ Neurodegenerative Disorder Research Center, Division of Life Sciences and Medicine, University of Science and Technology of China, Hefei, Anhui, China; ^2^ Department of Neurology, Institute on Aging and Brain Disorders, The First Affiliated Hospital of USTC, Division of Life Sciences and Medicine, University of Science and Technology of China, Hefei, Anhui, China

## Abstract

**Background:**

Identifying reliable and sensitive biomarkers to track neurodegeneration and cognitive impairment in Alzheimer's Disease (AD) is crucial for staging the severity of the disease.

**Method:**

This study enrolled 435 participants from the China Aging and Neurodegenerative Disorder Initiative (CANDI) cohort from 2018 through 2023. Follow‐up imaging data and cognitive assessments was conducted for up to four years (median follow‐up time = 21 months). Participants included cognitively unimpaired (CU) individuals, those with mild cognitive impairment (MCI), AD, and non‐AD dementia (non‐ADD). CSF levels of NPTX1 and NPTXR were measured and analyzed for their associations with cognitive function scores, cortical thickness, brain atrophy and cognitive decline. Their ability to differentiate between progressive and stable MCI was also assessed.

**Result:**

Here we show that, lower cerebrospinal fluid (CSF) levels of NPTX1 and its receptor, NPTXR, were associated with more severe cognitive impairment in the AD continuum. We also found that NPTX1 and NPTXR were positively associated with vertex‐wise cortical thickness across in all individuals, particularly in those with Aβ pathology. Moreover, lower levels of NPTXR and NPTX1 were associated with faster rates of longitudinal brain atrophy and cognitive decline. Furthermore, baseline levels of CSF NPTXR (AUC = 0.808) and NPTX1 (AUC = 0.811) could effectively predict progression to clinical dementia over a 3‐year period in mild cognitive impairment participants.

**Conclusion:**

CSF NPTXs are promising biomarkers for staging neurodegeneration and cognitive dysfunction in the AD continuum, offering valuable insights for personalized therapeutic interventions and clinical trial optimization.

